# Characterization of gene expression profiles in Alzheimer’s disease and osteoarthritis: A bioinformatics study

**DOI:** 10.1371/journal.pone.0316708

**Published:** 2025-02-07

**Authors:** Nian Liu, Qian Deng, Zining Peng, Danning Mao, Yuanbo Huang, Fanyu Meng, Xiaoyu Zhang, Jiayan Shen, Zhaofu Li, Weitian Yan, Jiangyun Peng

**Affiliations:** 1 First School of Clinical Medicine, Yunnan University of Chinese Medicine, Kunming, Yunnan, PR China; 2 Department of Rheumatology, The No.1 Affiliated Hospital of Yunnan University of Chinese Medicine, Kunming, Yunnan, PR China; Columbia University Irving Medical Center, UNITED STATES OF AMERICA

## Abstract

**Background:**

Alzheimer’s disease (AD) and Osteoarthritis (OA) have been shown to have a close association in previous studies, but the pathogenesis of both diseases are unclear. This study explores the potential common molecular mechanisms between AD and OA through bioinformatics analysis, providing new insights for clinical treatment strategies.

**Methods:**

The AD and OA-related datasets were downloaded from the gene expression database GEO. The datasets were analyzed to obtain differentially expressed gene (DEG) datasets for OA and AD, respectively. The intersection of these DEGs was analyzed to identify common DEGs (Co-DEGs). Subsequently, the Co-DEGs were enriched, and a protein-protein interaction network was constructed to identify core genes. The expression of these genes was validated in a separate dataset, and their diagnostic value for the diseases was analyzed. In addition, the core genes were analyzed using gene set enrichment analysis and single-gene genome variation analysis.

**Results:**

Analysis of DEGs on gene chips from OA and AD patients revealed significant changes in gene expression patterns. Notably, EFEMP2 and TSPO, genes associated with inflammatory responses, showed lower expression levels in both AD and OA patients, suggesting a downregulation in the pathological backgrounds of these diseases. Additionally, GABARAPL1, which is crucial for the maturation of autophagosomes, was found to be upregulated in both conditions. These findings suggest the potential of these genes as diagnostic biomarkers and potential therapeutic targets. However, to confirm the effectiveness of these genes as therapeutic targets, more in-depth mechanistic studies are needed in the future, particularly to explore the feasibility and specific mechanisms of combating disease progression by regulating the expression of these genes.

**Conclusions:**

This study suggests that AD and OA shares common molecular mechanisms. The identification of EFEMP2, GABARAPL1, and TSPO as key target genes highlights potential common factors in both diseases. Further investigation into these findings could lead to new candidate targets and treatment directions for AD and OA, offering promising avenues for developing more effective and targeted therapeutic interventions.

## Introduction

Alzheimer’s disease (AD) and osteoarthritis (OA) are significant age-related public health challenges, both characterized by progressive degeneration and substantial impact on quality of life. AD, the most common neurodegenerative disease and main cause of cognitive impairment in the elderly [1], affects approximately 50 million people worldwide, with age being the primary risk factor [2]. The number of patients with AD has risen sharply, and it is estimated that there will be 150 million AD patients worldwide by 2050 [3]. Currently, it is believed that the pathological changes of AD mainly involve oxidative stress, mitochondrial dysfunction, neuroexcitatory toxicity, and neuroinflammation [4]. Its typical pathological features are extracellular amyloid-beta (Aβ) plaques and intracellular neurofibrillary tangles composed of hyperphosphorylated tau protein [5].

Similarly, OA, a prevalent musculoskeletal disorder, is characterized by the progressive degeneration of joint cartilage, leading to pain and functional impairment. Affecting approximately 7.6% of the global population, OA’s burden is expected to increase by 60% to 100% by 2050 [6]. The risk factors for OA include aging, genetics, joint injury or overuse, obesity, and others. These factors lead to gradual wear and tear of joint cartilage, ultimately resulting in inflammation and joint deformity [7].

An interesting phenomenon is the frequent co-occurrence of AD with skeletal diseases, notably OA [8–11]. Both conditions share similarities, including aging, inflammation, and overlapping risk factors, suggesting potential shared mechanisms. Therefore, some studies suggest that the brain and bone tissue can regulate each other through the bone-brain axis in various ways [1]. Observational studies, as summarized in Table 1, have revealed that patients with OA exhibit a significantly increased risk of developing AD, potentially due to factors such as mood disturbances, pain, and sleep disorders. Experimental studies have further confirmed that the simultaneous presence of OA and AD may exacerbate inflammation, Aβ deposition, and cognitive impairment [12–14]. For instance, persistent peripheral inflammation in OA is associated with elevated levels of inflammatory mediators, such as IL-1β, IL-6, and TNF [15, 16], which can cross the blood-brain barrier or be produced by glial cells, leading to hyperphosphorylation of tau and neuronal loss in AD [17–20]. Although some experts have suggested that the low-grade systemic inflammation induced by OA, as well as the faster accumulation of Aβ and the higher Aβ-dependent future tau protein deposition in the primary motor and somatosensory regions, may increase the risk of AD [21–23], a recent Mendelian randomization study based on individuals of European descent did not find a causal relationship between OA and AD [24]. This finding may be due to the homogeneity of the study population in terms of genetic, environmental, or other relevant factors. Homogeneity can be a limitation in such studies because it may mask subtle associations or causal links that might be detectable in a more diverse population. Therefore, there is a need to conduct the analysis in a broader context.

**Table 1:** Current observational studies on Alzheimer’s disease (AD) and osteoarthritis (OA).

Research subject	Region	Age	Result	References
35,149 patients with OA 70,298 patients without OA	Taiwan, China	≥18 years old	Individuals with OA have a higher risk of developing dementia compared to those without OA.	[25]
2,478 patients with OA 19,004 patients without OA	U.S.	≥40 years old	OA and related joint pain are closely associated with perceived memory loss.	[26]
6,126 patients with OA 18,883 patients without OA	U.S.	≥65 years old	The presence and absence of pain interference are both significantly associated with an increased risk of AD related dementias. Additionally, nonsteroidal anti-inflammatory drugs are associated with a reduced risk of cognitive impairment and dementia.	[27]
4,545 patients with OA 12,389 patients without OA	U.S.	≥65 years old	OA is associated with an increased risk of AD related dementias, and this association is particularly evident in patients with OA and pain.	[28]
79 patients with OA 293 patients without OA	-	OA patients: (74.2 ± 6.22), Non-OA patients: (74.8 ± 5.36)	Compared to individuals without OA, the HpVR (hippocampal/intracranial volume x 10^3^) of OA patients decreased more rapidly over time, after controlling for other potential confounding factors (including age, education level, gender, and APOE4 genotype).	[29]
119 patients with OA 255 patients without OA	-	OA patients: 76 (median) Non-OA patients: 75 (median)	In OA patients, Aβ deposition occurs more rapidly, and it induces higher Aβ-related tau deposition in the primary motor and somatosensory cortices of Aβ-positive elderly individuals.	[23]
63,081 patients with OA 403,379 patients without OA	-	OA patients: 56.12(8.14) Non-OA patients: 60.70(6.37)	OA may increase the risk of dementia, while OA treatments (surgery and medication) can lower the risk of dementia. In particular, nonsteroidal anti-inflammatory drugs and opioids have a significant protective effect against dementia. Furthermore, OA is largely associated with a reduction in the area of gray matter.	[30]

Another analysis revealed a relationship between the expression of certain genes in knee osteoarthritis and the release of peripheral inflammatory factors, hyperphosphorylation of tau protein, and activation of astrocytes [31]. However, the specific connection between AD and OA remains unclear. Therefore, we aim to explore the bidirectional relationship between them by conducting a bioinformatics joint analysis of gene chips for both OA and AD. We will delve into the biological processes of the overlapping genes between these two diseases, with the goal of understanding the common pathogenic mechanisms underlying AD and OA. This could open up new avenues for treatment strategies or more personalized therapies for these two diseases.

## Materials and methods

### Data collection and differential analysis

**Fig 1:**
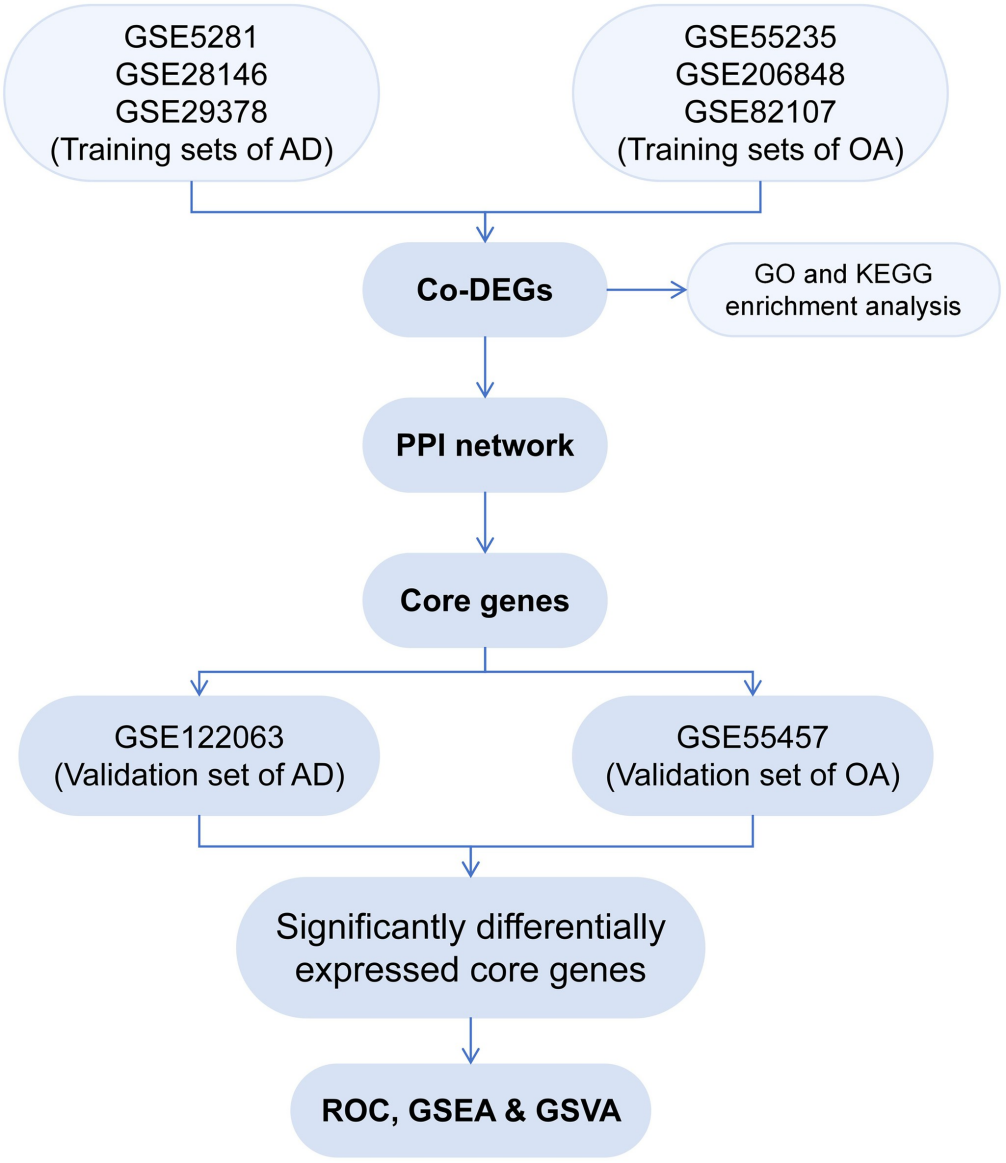
Overview of study design. AD: alzheimer’s disease; OA: osteoarthritis; Co-DEGs: common differentially expressed genes; GO: gene ontology; KEGG: kyoto encyclopedia of genes and genomes; PPI: protein-protein interaction; ROC: receiver operating characteristic; GSEA: gene set enrichment analysis; GSVA: single-gene genome variation analysis.

Fig 1 illustrates the study flowchart. The datasets pertaining to AD and OA were acquired by querying the GEO database (https://www.ncbi.nlm.nih.gov/geo/) with the keywords ’Alzheimer’s disease’ or ’osteoarthritis’. The datasets were selected based on the following inclusion criteria: 1) samples must be from human subjects; 2) the studies must include both control and disease groups; 3) sample types should be consistent across studies; and 4) datasets should have complete platform information available. Exclusion criteria included: 1) non-human gene chips; and 2) incomplete platform information. The gene probes in the original data were annotated using the annotation files of each chip platform, and the probes without matching gene names were excluded. If multiple different probes detected the same gene, the mean of these probes was taken as the final expression level of the gene. After standardizing the expression levels of each gene in each group, the merged training set was batch-corrected using the "sva" and "limma" packages in R (v4.3.0). Specifically, the "sva" package was first employed to identify and estimate latent variables associated with batch effects, which were then adjusted to minimize their interference with the data. Subsequently, the "limma" package was used for linear model fitting to further refine the data and assess changes in gene expression levels. Based on the corrected data, we screened for differentially expressed genes (DEGs) using criteria of adj. *P* < 0.05 and |log_2_ (FC)| > 0.5. The threshold of adj. *P* < 0.05 was chosen as it is a commonly accepted threshold for statistical significance in genomic studies, providing a control for the false discovery rate. The |log_2_(FC)| > 0.5 threshold was selected to focus on genes with at least a modest fold change, which, is likely to be biologically meaningful while excluding genes with very small changes that might be due to noise or technical variability. The intersection of the up- and down-regulated DEGs in the two diseases was taken.

Additionally, it should be noted that all data used in this study were de-identified and publicly available from the GEO database, therefore ethical approval for data use was not required. The use of these datasets adheres to the policies and guidelines set by the GEO database regarding data access and usage.

### Enrichment analysis

Using the "clusterProfiler" package in R (v4.3.0), with a filtering condition of *P*<0.05, the common DEGs (Co-DEGs) of the 2 disease groups were analyzed for gene ontology (GO), and Kyoto Encyclopedia of Genes and Genomes (KEGG). To ensure the accuracy of the enrichment results, multiple testing correction was applied to control the false discovery rate (FDR). Specifically, the enrichGO function was used for GO enrichment analysis, and the enrichKEGG function was used for KEGG enrichment analysis. Both functions internally correct *P* values to account for multiple testing. For visualization, the barplot and dotplot functions were used to generate intuitive bar charts and bubble plots that effectively present the enrichment results for both GO and KEGG analyses.

### Identification and expression of core genes

The Co-DEGs were input into the STRING (https://cn.string-db.org/) database, with species set to "Homo sapiens", and the minimum interaction threshold set to the highest confidence level of "highest confidence (0.4)", to obtain a Protein-Protein Interaction (PPI) network. Then, the PPI network was visualized using the Cytoscape v.3.8.0, and the top 10 core genes were ranked based on degree centrality. At the same time, the association between the core genes and the two diseases (OA and AD) was further evaluated using external validation sets. These sets comprised gene data from independent cohorts that met the inclusion criteria established in our method. The core gene expression boxplot was drawn using the "ggpubr" package in R (v 4.3.0) to visualize the expression levels of the core genes across different samples, facilitating comparisons between the diseases and controls.

### Diagnostic value of core genes

The diagnostic association between core genes and the disease was further validated using a disease-control set model. First, the "pROC" package in R (v4.3.0) was used to generate Receiver Operating Characteristic (ROC) curves for each core gene. The area under the curve (AUC), ranging from 0 to 1, was employed as a metric to assess the diagnostic accuracy of the core genes, where an AUC value closer to 1 indicates a better diagnostic performance. To evaluate the robustness and stability of our predictive model, a logistic regression model was constructed using the core genes as predictors. The model’s performance was then assessed through ROC analysis with internal cross-validation, and the resulting AUC, along with its 95% confidence interval (CI) were reported. Additionally, violin plots of the gene expressions were created using the "ggpubr" package to visually compare the expression levels between the disease and normal groups.

### Core genes enrichment analysis

To study the signaling pathways associated with core genes, we conducted Gene Set Enrichment Analysis (GSEA) to assess the enrichment of these genes within the training set data. Then, the gene set obtained was compared with a predefined KEGG signaling pathway set to evaluate its enrichment level, with "c2.cp.kegg.symbols.gmt" serving as the reference genome. Meanwhile, to investigate the impact of core genes on KEGG pathways, we performed Gene Set Variation Analysis (GSVA). We used the "limma" software package to compare the GSVA scores of disease samples and normal samples. Significance was determined by the criteria for *|t*| > 2 and *P* < 0.05.

## Results

### GEO dataset grouping and expression

**Fig 2:**
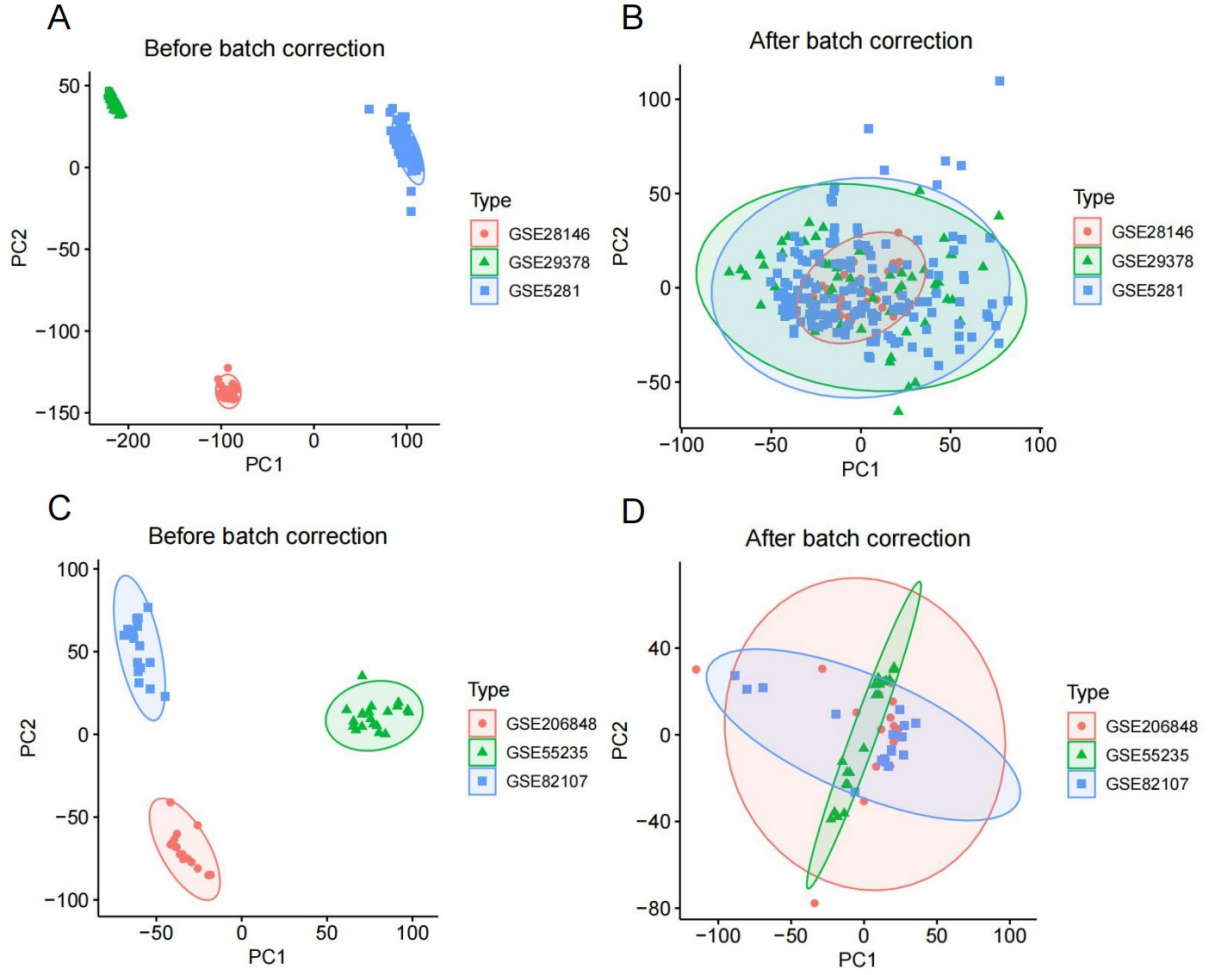
Sample distribution principal component analysis (PCA) plot. PCA plot of sample distribution before (A) and after (B) of the AD-related training set. PCA plot of sample distribution before (C) and after (D) of the OA-related training set.

**Fig 3:**
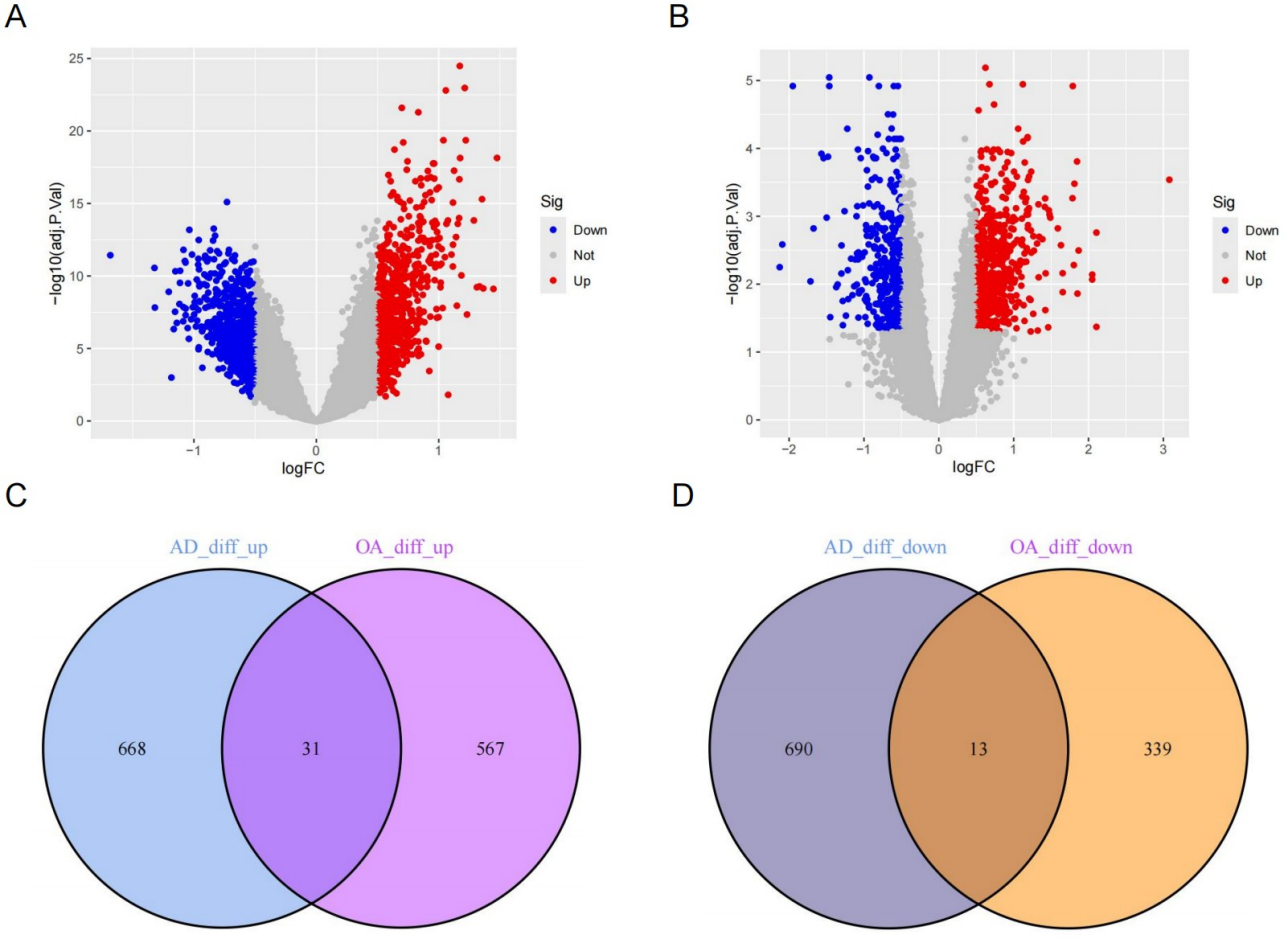
Differential expression analysis. Gene expression in the AD training group (A) and OA training group (B) between disease samples and normal samples. (C) The overlap of upregulated DEGs in AD and OA training sets. (D) The overlap of downregulated DEGs in AD and OA training sets.

The AD-related GSE5281, GSE28146, GSE29378, and GSE122063 datasets were obtained, with GSE5281, GSE28146, and GSE29378 serving as the training set and GSE122063 as the external validation set. The OA-related GSE55235, GSE55457, GSE206848, and GSE82107 datasets were obtained, with GSE55235, GSE206848, and GSE82107 serving as the training set and GSE55457 as the external validation set. The normalized gene expression profile can be found in S1 and S2 Tables. The detailed information of the two gene chip datasets is shown in Table 2. We performed batch processing of the training set gene data (Fig 2). Subsequently, 1402 DEGs were identified from the AD gene chip, including 703 down-regulated genes and 699 up-regulated genes (Fig 3A, S3 Table). 950 DEGs were identified in the OA gene chip, including 352 down-regulated genes and 598 up-regulated genes (Fig 3B, S3 Table). There were 31 up-regulated Co-DEGs between AD and OA (Fig 3C), and 13 down-regulated Co-DEGs (Fig 3D).

**Table 2:** Basic information and grouping of GEO data set.

GEO ID	Platform File	Control/Example			Disease/Example			Grouping	Disease	Tissue
		case	Age	sex (male/female)	case	Age	sex (male/female)			
GSE5281	PL570	74	-	53/21	87	-	50/37	Training set	AD	brain
GSE28146	PL570	8	86.38±7.85	6/2	22	86.27±7.85	6/16	Training set	AD	brain
GSE29378	GPL6947	32	81.65±6.77	22/10	31	76.64±8.96	16/15	Training set	AD	brain
GSE122063	GPL16699	44	78.82±8.53	20/24	56	81.00±6.61	12/44	Validation set	AD	brain
GSE55235	GPL96	10	-	-	10	-	-	Training set	OA	synovial
GSE206848	GPL570	7	-	-	7	-	-	Training set	OA	synovial
GSE82107	GPL570	7	-	-	10	-	-	Training set	OA	synovial
GSE55457	GPL96	10	51.00±19.71	6/2	10	72.40±5.93	2/8	Validation set	OA	synovial

AD: Alzheimer’s disease; OA: Osteoarthritis

PL570: [HG-U133_Plus_2] Affymetrix Human Genome U133 Plus 2.0 Array

GPL6947: Illumina HumanHT-12 V3.0 expression beadchip

GPL16699: Agilent-039494 SurePrint G3 Human GE v2 8x60K Microarray 039381 (Feature Number version)

GPL96: [HG-U133A] Affymetrix Human Genome U133A Array

GPL570: [HG-U133_Plus_2] Affymetrix Human Genome U133 Plus 2.0 Array

### Enrichment analysis of Co-DEGs

**Fig 4:**
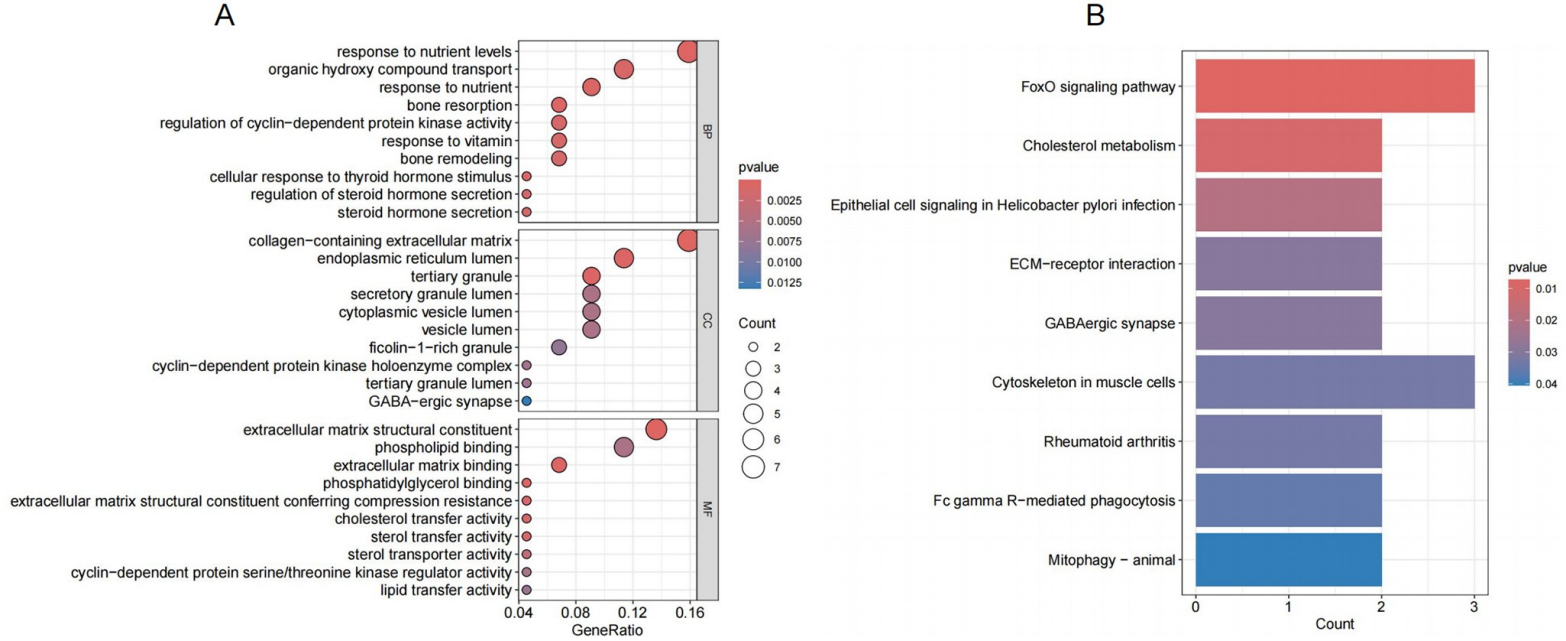
Gene ontology (GO) and Kyoto Encyclopedia of Genes and Genomes (KEGG) enrichment analyses were conducted on common DEGs (Co-DEGs) shared between AD and OA. (A) GO enrichment analysis of Co-DEGs. The x-axis represents the gene ratio (the proportion of DEGs in the GO term relative to the total), the y-axis represents the GO terms ordered by enrichment significance (with color scale indicating the significance level), and the size of the circles represents the number of enriched genes in each term. (B) KEGG enrichment analysis of Co-DEGs, with the x-axis showing the number of enriched genes and the y-axis represents the different KEGG pathways.

The 44 Co-DEGs between AD and OA were subjected to GO and KEGG enrichment analysis. The GO functional enrichment analysis revealed 1,246 functional categories. These were mainly mediated by affecting 226 biological processes (BPs), such as response to nutrient levels, response to nutrient, and organic hydroxy compound transport. The 43 molecular functions (MFs) mainly involved extracellular matrix structural constituent, phospholipid binding, and extracellular matrix binding. The 45 cellular components (CCs) were mainly located in collagen-containing extracellular matrix, endoplasmic reticulum lumen, and tertiary granule (Fig 4A, S4 Table).

The KEGG enrichment analysis yielded 10 related signaling pathways, mainly concentrated on FoxO signaling pathway, cholesterol metabolism, and epithelial cell signaling in helicobacter pylori infection (Fig 4B, S4 Table).

### Validation of core genes

**Fig 5:**
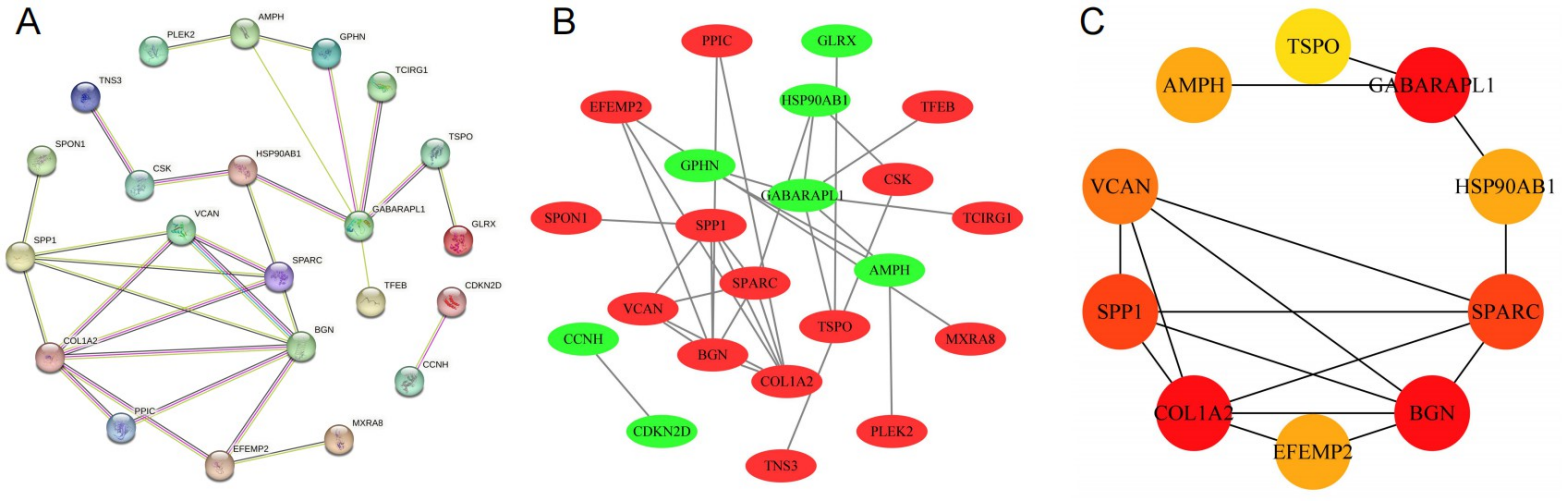
Protein-Protein Interaction (PPI) network. (A) PPI network of Co-DEGs. (B) Cytoscape v.3.8.0 facilitates the visualization of PPI networks. Green represents down-regulated Co-DEGs, while red represents up-regulated Co-DEGs. (C) PPI network of Co-DEGs in the degree algorithm; the color of the circles in the fig from yellow to red represents the gradual increase in the score.

**Fig 6:**
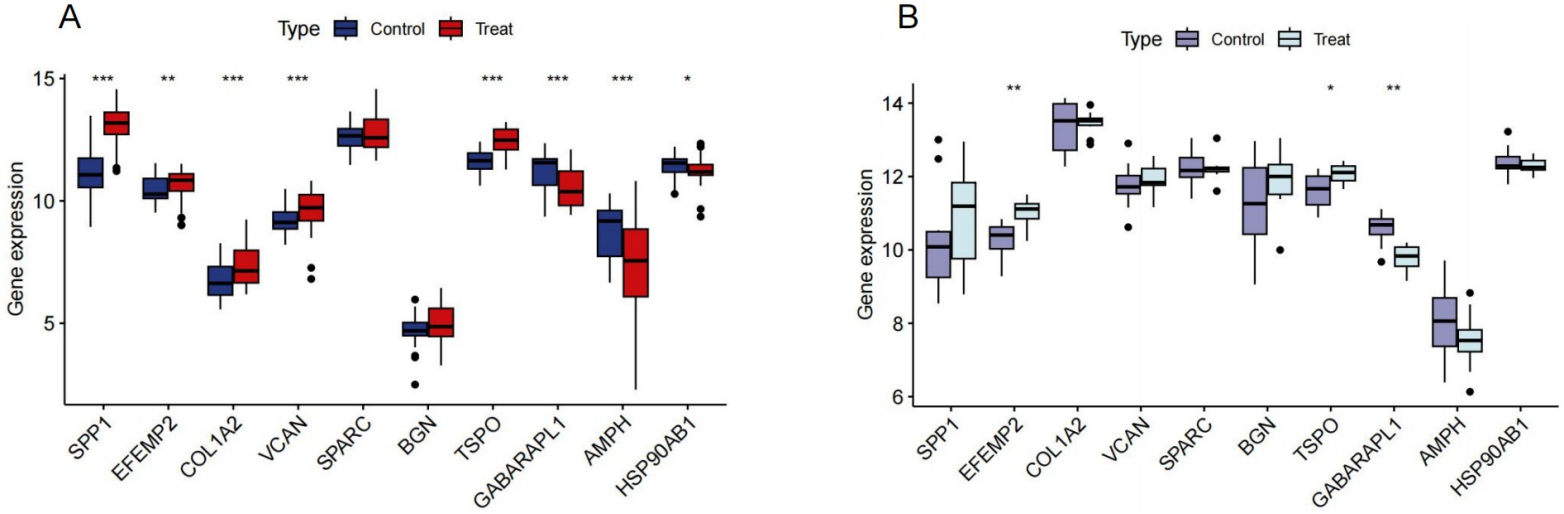
The top 10 CO-DEGs were identified based on their degree in the dataset. (A) Expression of the 10 core genes in the validation set of AD. (B) Expression of the 10 core genes in the validation set of OA. *P <0.05, **P < 0.01, ***P< 0.001.

The 44 Co-DEGs were used to construct a PPI interaction network, including 22 nodes (7 down-regulated and 15 up-regulated nodes), and 29 edges (Fig 5A and 5B). SPP1, EFEMP2, COL1A2, VCAN, SPARC, BGN, TSPO, GABARAPL1, AMPH, and HSP90AB1 were identified as the core genes associated with the two diseases based on their degree values (Fig 5C, S5 Table). Simultaneously, in the external validation for AD and OA, EFEMP2, GABARAPL1, and TSPO were found to be differentially expressed between the normal and disease group (*P* < 0.05) (Fig 6).

### The diagnostic value of core genes

**Fig 7:**
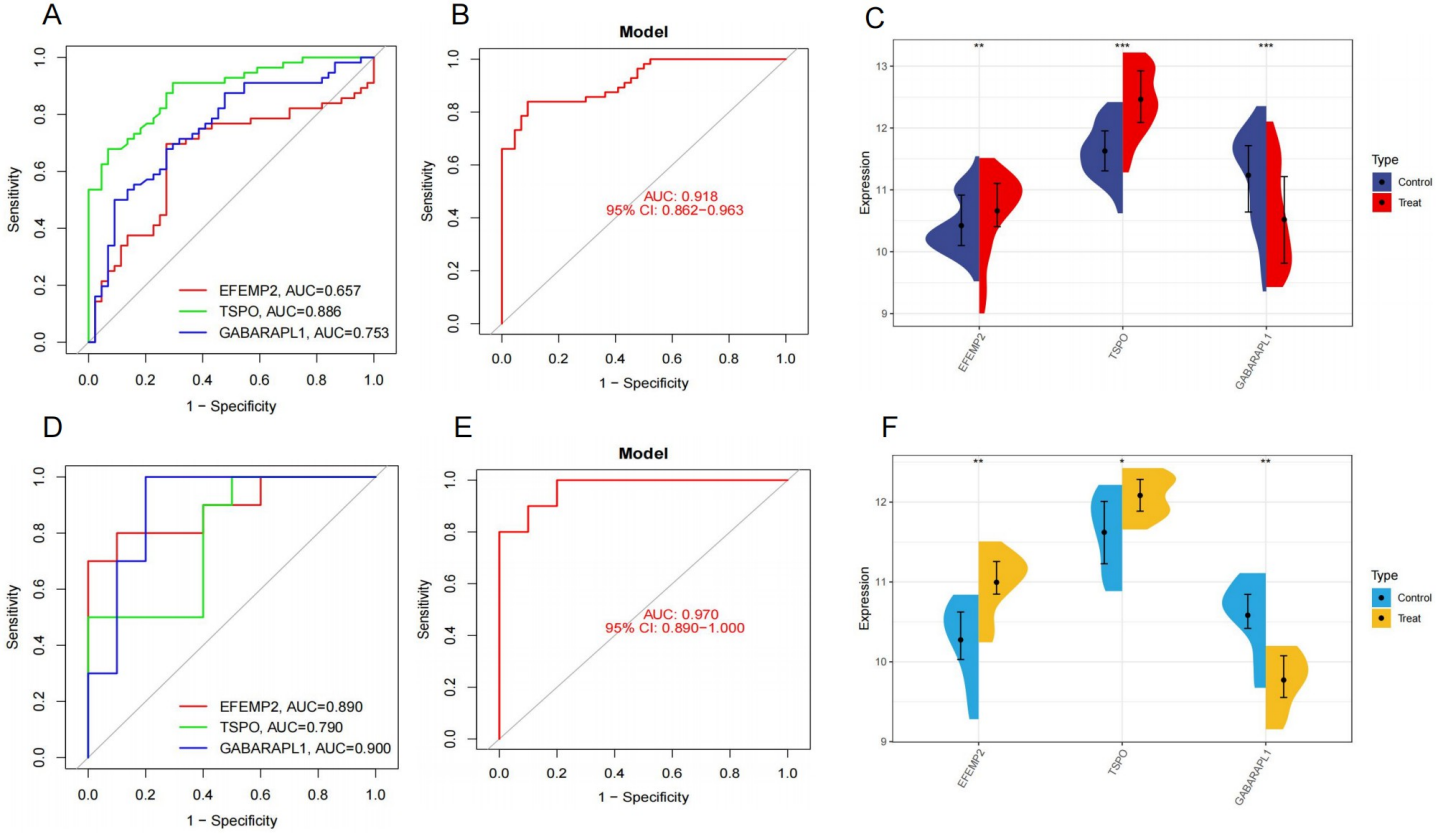
(A-B) Receiver Operating Characteristic (ROC) curves and model performance for significantly differentially expressed core genes (EFEMP2, GABARAPL1, TSPO) in AD validation. (C) Violin plot of EFEMP2, GABARAPL1, TSPO expression in AD validation. (D-E) ROC curves and model performance for EFEMP2, GABARAPL1, TSPO in OA validation. (F) Violin plot of EFEMP2, GABARAPL1, TSPO expression in OA validation.

In the external validation set for AD, the AUC values for EFEMP2, GABARAPL1, and TSPO were 0.657, 0.886, and 0.753 respectively (Fig 7A), all exceeding the threshold of 0.60, indicating moderate to good diagnostic accuracy. It is important to note that the logistic regression model’s AUC for AD was 0.918 with a 95% CI of 0.862 to 0.963 (Fig 7B), and while the AUC values for the individual genes were not all within this CI, they still demonstrated significant diagnostic potential. Moreover, their expression levels in the AD group were significantly different from those in the normal group (*P* < 0.01), as shown in Fig 7C. In the external validation set for OA, the AUC values for EFEMP2, GABARAPL1, and TSPO were 0.890, 0.790, and 0.900 respectively (Fig 7D), all exceeding the threshold of 0.70, indicating good diagnostic accuracy. The logistic regression model’s AUC for OA was 0.970 with a 95% CI of 0.890 to 1.000 (Fig 7E), and similar to the AD analysis, while the individual gene AUCs were not all within this CI, they still showed promise as diagnostic markers. Their expression levels also exhibited significant disparity when compared to the normal group (*P* < 0.05) (Fig 7F). However, it is important to note that while these genes show promise as diagnostic markers for each disease, our study did not assess their ability to differentiate between AD and OA specifically.

### Enrichment analysis of core genes

**Fig 8:**
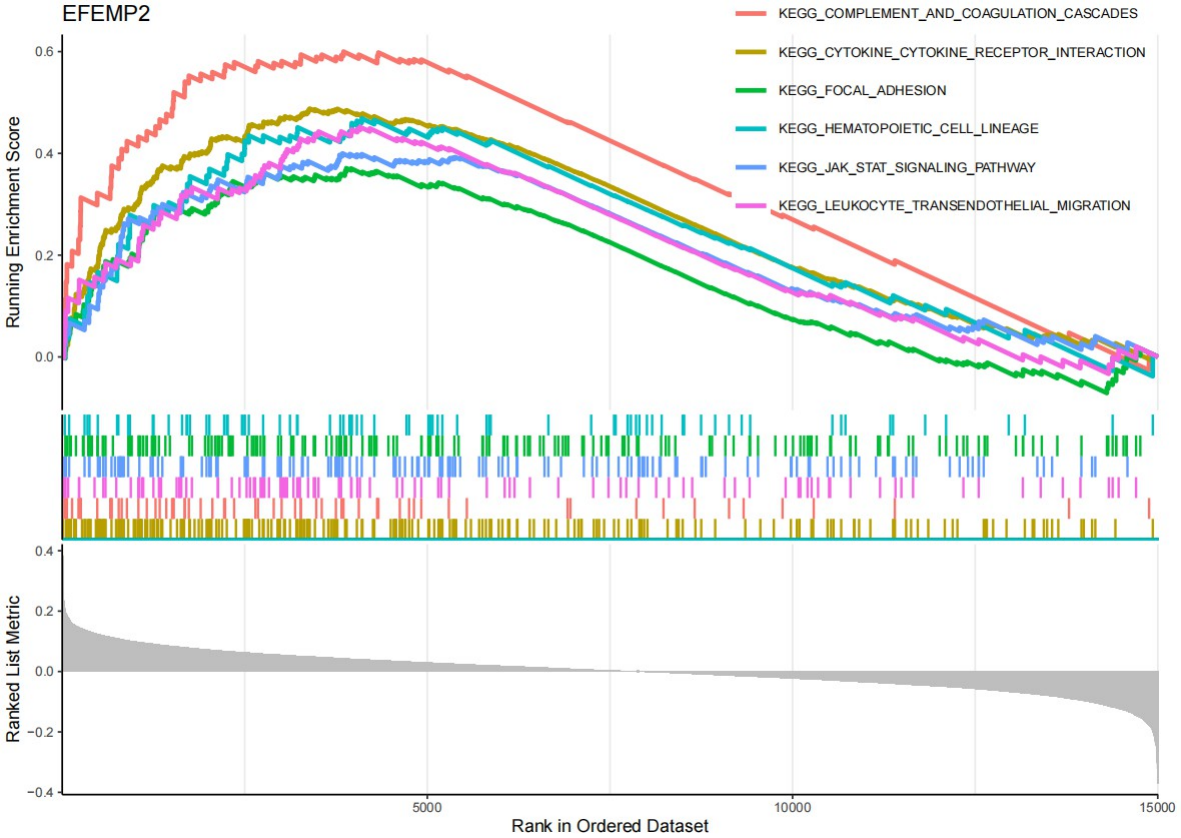
Gene set enrichment analysis (GSEA) of EFEMP2 in AD. y-axis: The "Running Enrichment Score" reflects the enrichment level of the EFEMP2, GABARAPL1, and TSPO gene sets in AD. The "Ranked List Metric" displays the ranking of genes within the dataset. x-axis: Represents the position of genes in the sorted dataset.

**Fig 9:**
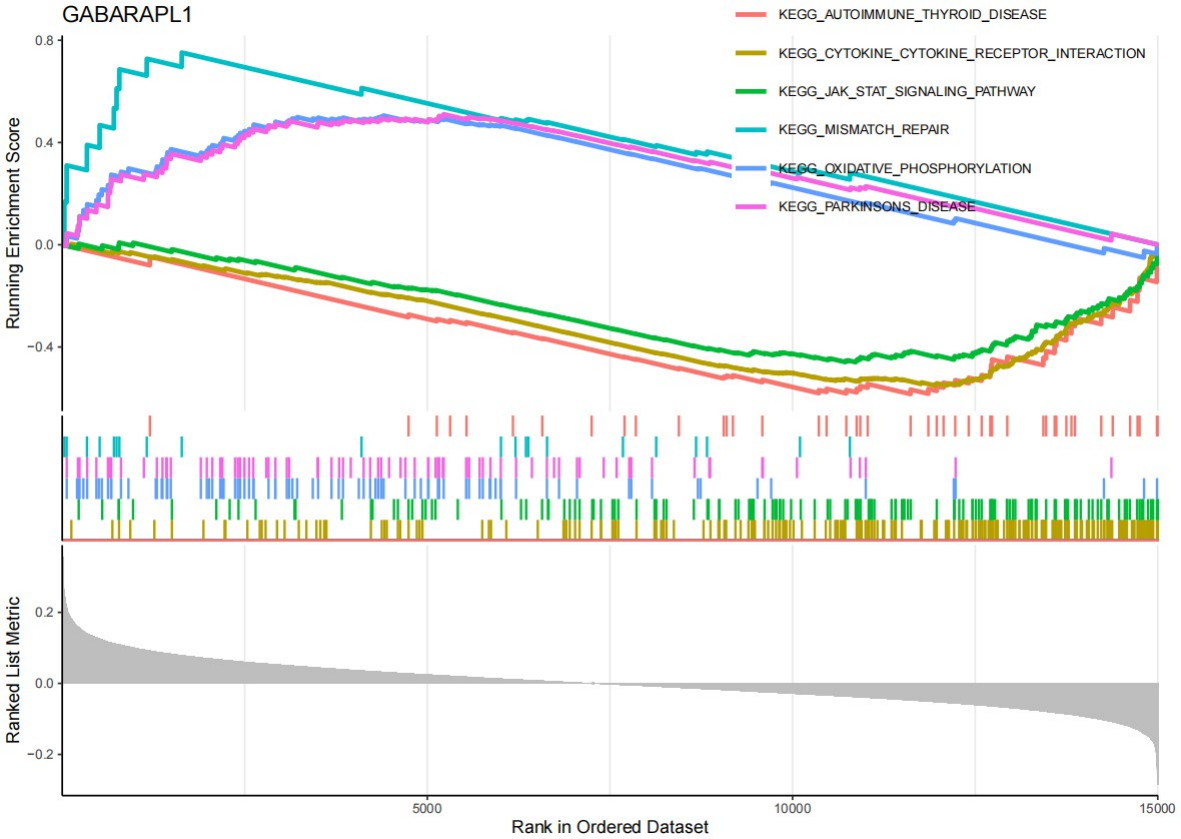
Gene set enrichment analysis (GSEA) of GABARAPL1 in AD. y-axis: The "Running Enrichment Score" reflects the enrichment level of the EFEMP2, GABARAPL1, and TSPO gene sets in AD. The "Ranked List Metric" displays the ranking of genes within the dataset. x-axis: Represents the position of genes in the sorted dataset.

**Fig 10:**
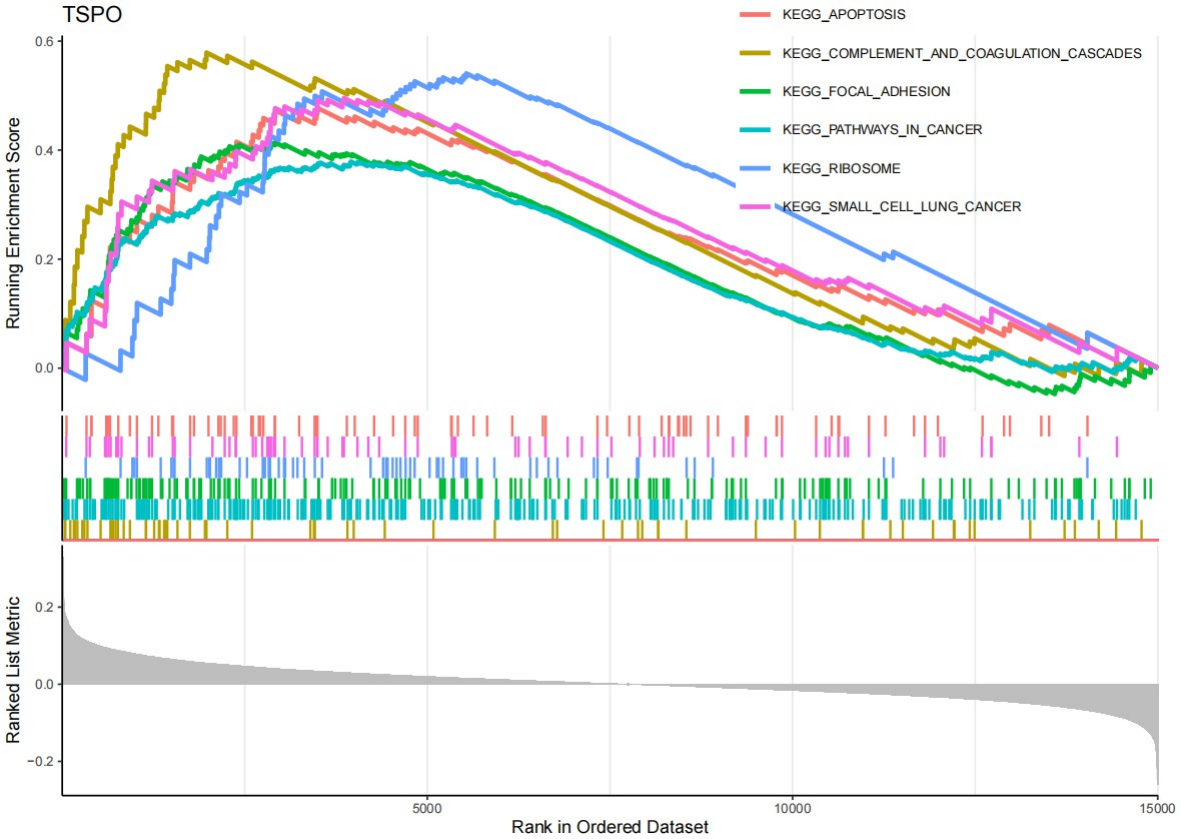
Gene set enrichment analysis (GSEA) of TSPO in AD. y-axis: The "Running Enrichment Score" reflects the enrichment level of the EFEMP2, GABARAPL1, and TSPO gene sets in AD. The "Ranked List Metric" displays the ranking of genes within the dataset. x-axis: Represents the position of genes in the sorted dataset.

The GSEA enrichment analysis results (Fig 8, S6 Table) show that in AD, EFEMP2’s high expression is mainly associated with inflammation and immune responses, such as complement and coagulation cascades, cytokine-cytokine receptor interaction, focal adhesion, hematopoietic cell lineage, jak-stat signaling pathway, and leukocyte transendothelial migration. Through GSVA analysis (S1 Fig), we identified that the upregulation of EFEMP2 expression is implicated in several critical biological processes, indicating its potential essentiality for maintaining cellular metabolic equilibrium, facilitating DNA repair, ensuring genomic stability, mediating cell signal transduction, and enhancing antioxidant defense mechanisms. The GSEA findings (Fig 9, S2 Fig, S7 Table) indicate that GABARAPL1 is significantly enriched in immune-related pathways in AD, encompassing pathways such as autoimmune thyroid disease and cytokine-cytokine receptor interaction. Furthermore, GABARAPL1 is implicated in the JAK-STAT signaling pathway, which previous studies have identified as an important component in the pathogenesis of AD [32, 33]. The GSEA results (Fig 10, S8 Table) for TSPO suggest that this gene may be closely associated with the apoptosis pathway, as well as the complement and coagulation cascades. Additionally, it may also have certain connections with the focal adhesion pathway and cancer-related processes. The GSVA findings (S3 Fig) indicate that the downregulation of the TSPO gene in AD is linked to pathological mechanisms such as inflammatory responses, neurodegeneration, metabolic disorders, and immune abnormalities.

**Fig 11:**
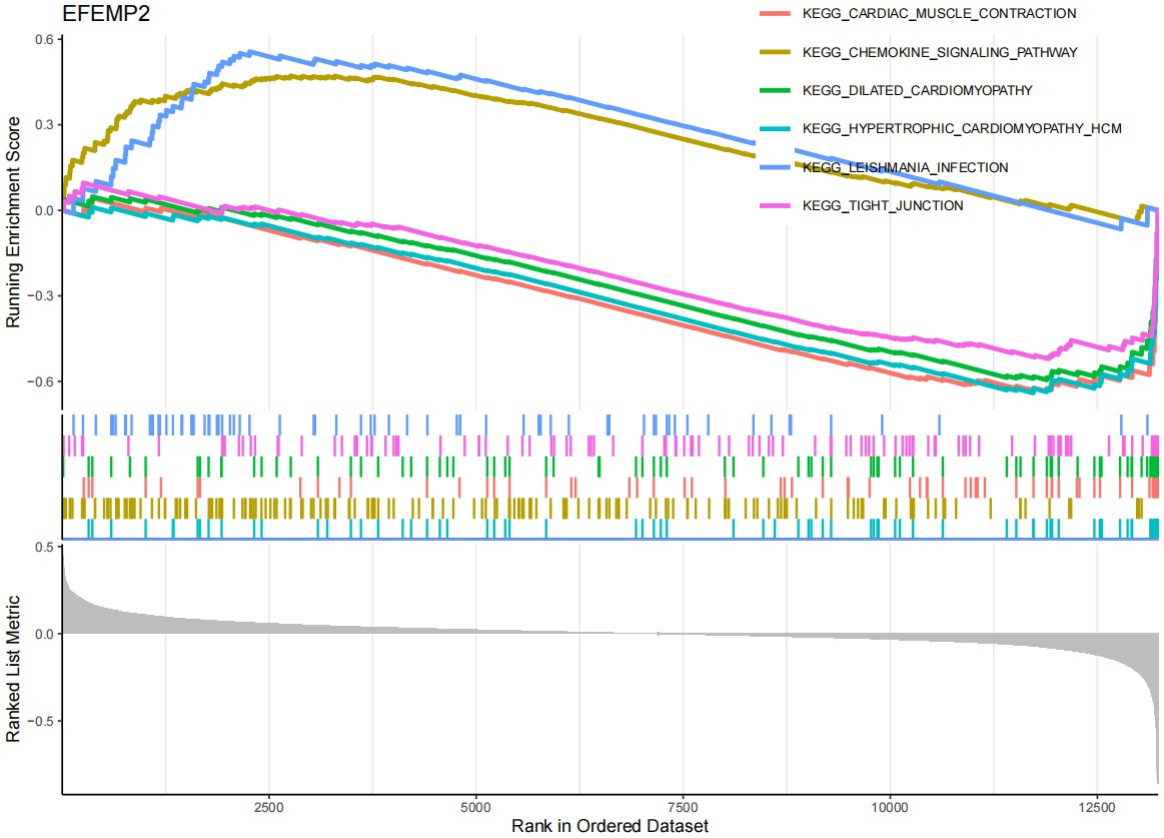
GSEA of EFEMP2 in OA. y-axis: The "Running Enrichment Score" reflects the enrichment level of the EFEMP2, GABARAPL1, and TSPO gene sets in OA. The "Ranked List Metric" displays the ranking of genes within the dataset. x-axis: Represents the position of genes in the sorted dataset.

**Fig 12:**
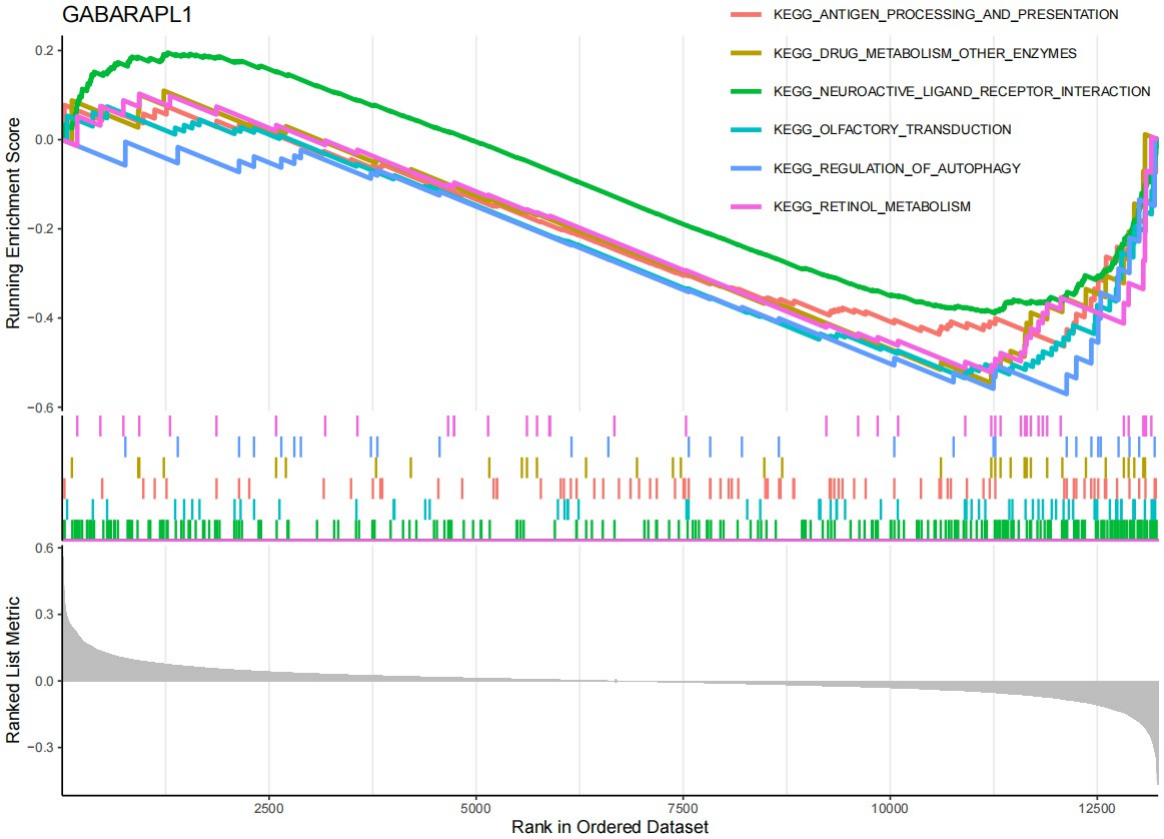
GSEA of GABARAPL1 in OA. y-axis: The "Running Enrichment Score" reflects the enrichment level of the EFEMP2, GABARAPL1, and TSPO gene sets in OA. The "Ranked List Metric" displays the ranking of genes within the dataset. x-axis: Represents the position of genes in the sorted dataset.

**Fig 13:**
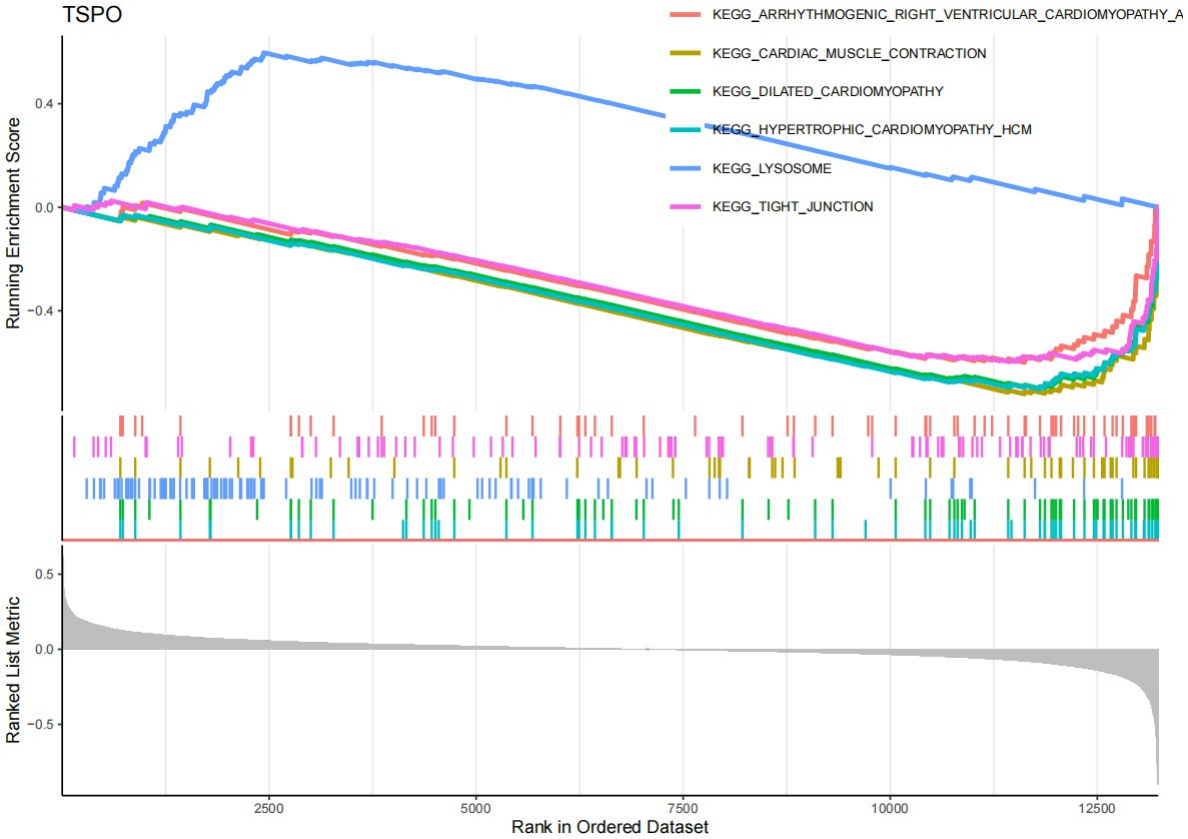
GSEA of TSPO in OA. y-axis: The "Running Enrichment Score" reflects the enrichment level of the EFEMP2, GABARAPL1, and TSPO gene sets in OA. The "Ranked List Metric" displays the ranking of genes within the dataset. x-axis: Represents the position of genes in the sorted dataset.

On the other hand, in the GSEA and GSVA analysis of OA (Fig 11, S4 Fig, S9 Table), the EFEMP2 gene was upregulated in the cartilage tissue of OA patients, and was associated with multiple pathways and processes related to OA, including cardiac muscle contraction, chemokine signaling pathway, dilated cardiomyopathy, among others. This suggests that the EFEMP2 gene may participate in the development of OA by promoting inflammatory responses, affecting chondrocyte function and modulating immune cell function. The GABARAPL1 gene (Fig 12, S5 Fig, S10 Table) may affect immune responses, drug metabolism, neural signal transmission, and autophagy processes in OA, thereby promoting the development of OA. However, it is interesting that no upregulated pathways of EFEMP2 and GABARAPL1 were enriched in GSVA. The reason for this may be the influence of study sample selection and handling on the results, such as sample size, disease subtype, gender, age, etc., which may affect gene expression and pathway enrichment. The GSEA results (Fig 13, S11 Table) show that the TSPO gene may be associated with various cardiovascular diseases in the context of OA, including Arrhythmogenic Right Ventricular Dysplasia (ARVD), Cardiomyopathy with Conduction Disorders (CMC), Dilated Cardiomyopathy (DCM), and Hypertrophic Cardiomyopathy (HCM). In addition, the TSPO gene may also be related to lysosomes and tight junctions. According to the GSVA results (S6 Fig), the downregulation of TSPO gene in OA is associated with multiple metabolic pathways and signaling pathways, including lysosomal pathway, glycosaminoglycan degradation pathway, apoptosis pathway, and inflammation pathway, etc. The results suggest that the TSPO gene may participate in apoptosis, inflammation, changes in chondrocytes, and metabolism, etc., thereby promoting the development of OA.

## Discussion

In this study, we analyzed the gene chip data of AD and OA using bioinformatics methods. Through GO enrichment analysis, we found that the Co-DEGs of AD and OA were related to various biological contexts related to extracellular matrix assembly, cell adhesion, nutrient metabolism, and signal transduction. Through KEGG pathway enrichment analysis, the FoxO signaling pathway was the most significantly enriched. This pathway is related to cell survival, growth, metabolism, and stress response regulation [34]. For AD and OA, this pathway may be related to regulating apoptosis, inflammation, and extracellular matrix degradation processes. For example, FOXO and TNF-α cause oxidative stress by excessive release of ROS. Oxidative stress leads to mitochondrial dysfunction and energy metabolism defects, causing significant inflammation, impairment of synaptic transmission function, and neuron death [35]. Overexpression of FOXO1, FOXO3, and FOXO4 can prevent age-related bone loss, and in addition, overexpression of FOXO1 also counteracts IL-1β [36, 37]. Cholesterol metabolism plays an important role in cell membrane structure, signal transduction, and neurotransmitter synthesis [38]. A study screened neurons derived from induced pluripotent stem cells of AD patients and identified cholesterol esters, which are storage products of excess cholesterol, as upstream regulators of tau in the early stages of AD [39]. In OA, the CH25H-CYP7B1-RORα axis in cholesterol metabolism in cartilage cells may be a key regulatory factor in the pathogenesis of OA [40]. Therefore, it can be seen that abnormal cholesterol metabolism in OA and AD may lead to cell dysfunction and neurodegeneration. The epithelial cell signaling in helicobacter pylori infection may be related to activating inflammatory responses, affecting neurotransmitter synthesis, and affecting the gut microbiome and immune system, which are implicated in AD and OA. Other pathways also play an important role in AD and OA, such as mitophagy—animal signaling pathway. Studies have proposed that autophagy may have a bidirectional relationship with Aβ and Tau [41], and autophagy also plays a significant role in balancing inflammation in AD [42]. In OA, autophagy is closely related to cartilage matrix metabolism and apoptosis [43].

Further research led us to identify EFEMP2, GABARAPL1, and TSPO as potential genes that are common to both AD and OA. EFEMP2 is essential for the formation of elastic fibers and the development of connective tissue, and serves as a negative regulator target of miR-211-5p. It is involved in chondrocyte differentiation and is associated with inflammatory cytokine expression [44], neuronal apoptosis [45], and tumor autophagy [46]. GABARAPL1 has been shown in mitochondrial stress conditions to promote the clearance of damaged mitochondria and play a key role in the maturation of autophagosomes [47, 48]. A study has found that inhibiting autophagy in OA exacerbates the degeneration of chondrocytes and cartilage [49], suggesting that GABARAPL1 may regulate autophagy in OA to influence its pathological changes. Cartilage, lacking blood vessels and nerves, heavily relies on autophagy to maintain internal homeostasis and clear damaged components. GABARAPL1, by regulating autophagy, may play a role in the turnover of extracellular matrix components and the modulation of inflammatory responses within the joint. Research suggests that GABARAPL1 may be a key gene in mitochondrial autophagy in AD, with a downregulated expression trend in AD [50], consistent with our findings. Alterations in GABARAPL1 expression can affect the efficiency of autophagy and contribute to the pathogenesis of neurodegenerative diseases. Meanwhile, in this study, GABARAPL1 was also enriched in the FoxO signaling pathway, and existing research has revealed its primary connection through autophagy. Nonetheless, research specifically exploring the role of autophagy in the common mechanisms underlying AD and OA remains limited. This finding suggests that the autophagy may play an interconnected role in the pathological progression of these two diseases.

TSPO has been extensively studied as a PET imaging biomarker for neuroinflammation and recently as a target for treating neurodegenerative diseases. TSPO ligands have been shown to potentially reduce toxic β-amyloid, reduce brain atrophy [51, 52], and improve mitochondrial autophagy in AD models [53]. The expression of the TSPO is significantly elevated in the synovial tissue of patients with OA and correlates with an increased level of the anti-inflammatory cytokine IL-10, a decreased level of the pro-inflammatory cytokine IL-8, and a reduction in pain intensity [54]. In this study, we found that both EFEMP2 and TSPO were upregulated in the disease group. Therefore, whether inhibiting the expression of these genes can reduce the risk of developing AD or OA is also a direction worthy of further investigation.

By synthesizing the results of GSEA and GSVA, we have gained insights into the potential involvement of EFEMP2, GABARAPL1, and TSPO in the pathogenesis of AD and OA within the "bone-brain axis," particularly in the contexts of inflammatory responses, cellular metabolism, immune regulation, and neurodegeneration. For instance, the balance of minerals such as calcium and phosphorus is not only critical for bone health but also directly impacts neural conduction and brain function [55]. Hormones like parathyroid hormone and calcitonin play pivotal roles in maintaining blood calcium homeostasis and neural function [56]. Aberrant fluctuations in these hormones can not only lead to bone diseases but may also impair cognitive function by affecting neuronal excitability and synaptic plasticity. Furthermore, sex hormones such as estrogen and testosterone are crucial for sustaining both bone health and brain function, and their level changes have been implicated in the increased risk of AD and OA [57, 58]. Notably, two key genes associated with inflammatory responses, EFEMP2 and TSPO, were found to be upregulated in the disease group. While these findings suggest potential roles for these genes in the disease process, whether their differential expression constitutes a causal factor in the pathogenesis of AD and OA or represents an ineffective counter-response remains to be determined.

## Conclusion

This study suggests that AD and OA may converge on shared molecular pathways, with EFEMP2, GABARAPL1, and TSPO emerging as pivotal target genes that play significant roles in inflammation, cellular metabolism, immune modulation, and neurodegenerative processes. Although our bioinformatics analysis has revealed potential links between these genes and protein networks associated with autophagy, we fully recognize that observational data alone are not sufficient to establish autophagy as a causal factor in the interplay between AD and OA. Therefore, these findings should be considered as a starting point, and further mechanistic studies are urgently needed to elucidate the exact roles of these genes and their associated processes in the pathogenesis of both diseases.

We acknowledge the limitations of our study, particularly the reliance on bioinformatics methods and publicly available datasets, which may introduce biases in data interpretation. To ensure the robustness and generalizability of our findings, replication in larger and more diverse populations is essential. Moreover, while our results provide biological plausibility for the involvement of these genes in AD and OA, contradictory evidence in the literature must be addressed through rigorous research. In particular, mechanistic studies are crucial to unravel the complex interactions between these genes, autophagy, and the development of AD and OA, ultimately guiding the development of more effective and precise therapeutic strategies.

## Supporting information

S1 Table Dataset normalized gene expression profiles for Alzheimer’s disease. (XLSX)

S2 Table Dataset normalized gene expression profiles for osteoarthritis. (XLSX)

S3 Table Differentially expressed genes in Alzheimer’s disease and osteoarthritis. (XLSX)

S4 Table GO and KEGG analysis results. (XLSX)

S5 Table Degree value of protein-protein interaction network of co-gengs. (XLSX)

S6 Table Gene set enrichment analysis of EFEMP2 in Alzheimer’s disease and osteoarthritis. (XLSX)

S7 Table Gene set enrichment analysis of GABARAPL1 in Alzheimer’s disease. (XLSX)

S8 Table Gene set enrichment analysis of TSPO in Alzheimer’s disease. (XLSX)

S9 Table Gene set enrichment analysis of EFEMP2 in Osteoarthritis. (XLSX)

S10 Table Gene set enrichment analysis of GABARAPL1 in Osteoarthritis. (XLSX)

S11 Table Gene set enrichment analysis of TSPO in Osteoarthritis. (XLSX)

S1 Fig Gene Set Variation Analysis (GSVA) of EFEMP2 in AD. The x-axis represents the *t*-value of the GSVA score, which measures the degree of difference in expression levels of the core gene set between the disease group and the control group. A larger absolute value of the *t*-value indicates a more significant difference between the two groups. The y-axis lists different signaling pathways, which are collections of interconnected molecular events in biology. In the figure, red represents a positive t-value, indicating that the expression level of the gene set in the disease group is upregulated compared to the control group; whereas green represents a negative *t*-value of the GSVA score, indicating that the expression level of the gene set in the disease group is downregulated compared to the control group.
(TIFF)

S2 Fig Gene Set Variation Analysis (GSVA) of GABARAPL1 in AD. The x-axis represents the *t*-value of the GSVA score, which measures the degree of difference in expression levels of the core gene set between the disease group and the control group. A larger absolute value of the *t*-value indicates a more significant difference between the two groups. The y-axis lists different signaling pathways, which are collections of interconnected molecular events in biology. In the figure, red represents a positive t-value, indicating that the expression level of the gene set in the disease group is upregulated compared to the control group; whereas green represents a negative *t*-value of the GSVA score, indicating that the expression level of the gene set in the disease group is downregulated compared to the control group.
(TIFF)

S3 Fig Gene Set Variation Analysis (GSVA) of TSPO in AD. The x-axis represents the *t*-value of the GSVA score, which measures the degree of difference in expression levels of the core gene set between the disease group and the control group. A larger absolute value of the *t*-value indicates a more significant difference between the two groups. The y-axis lists different signaling pathways, which are collections of interconnected molecular events in biology. In the figure, red represents a positive t-value, indicating that the expression level of the gene set in the disease group is upregulated compared to the control group; whereas green represents a negative *t*-value of the GSVA score, indicating that the expression level of the gene set in the disease group is downregulated compared to the control group.
(TIFF)

S4 Fig Gene Set Variation Analysis (GSVA) of EFEMP2 in OA. The x-axis represents the *t*-value of the GSVA score, which measures the degree of difference in expression levels of the core gene set between the disease group and the control group. A larger absolute value of the *t*-value indicates a more significant difference between the two groups. The y-axis lists different signaling pathways, which are collections of interconnected molecular events in biology. In the figure, red represents a positive t-value, indicating that the expression level of the gene set in the disease group is upregulated compared to the control group; whereas green represents a negative *t*-value of the GSVA score, indicating that the expression level of the gene set in the disease group is downregulated compared to the control group.
(TIFF)

S5 Fig Gene Set Variation Analysis (GSVA) of GABARAPL1 in OA. The x-axis represents the *t*-value of the GSVA score, which measures the degree of difference in expression levels of the core gene set between the disease group and the control group. A larger absolute value of the *t*-value indicates a more significant difference between the two groups. The y-axis lists different signaling pathways, which are collections of interconnected molecular events in biology. In the figure, red represents a positive t-value, indicating that the expression level of the gene set in the disease group is upregulated compared to the control group; whereas green represents a negative *t*-value of the GSVA score, indicating that the expression level of the gene set in the disease group is downregulated compared to the control group.
(TIFF)

S6 Fig Gene Set Variation Analysis (GSVA) of TSPO in OA. The x-axis represents the *t*-value of the GSVA score, which measures the degree of difference in expression levels of the core gene set between the disease group and the control group. A larger absolute value of the *t*-value indicates a more significant difference between the two groups. The y-axis lists different signaling pathways, which are collections of interconnected molecular events in biology. In the figure, red represents a positive t-value, indicating that the expression level of the gene set in the disease group is upregulated compared to the control group; whereas green represents a negative *t*-value of the GSVA score, indicating that the expression level of the gene set in the disease group is downregulated compared to the control group.
(TIFF)
