# Supplementary figures and images for: Characterization of gene expression profiles in Alzheimer’s disease and osteoarthritis: A bioinformatics study
Source: PLoS One. 2025 Feb 7;20(2):e0316708. doi: 10.1371/journal.pone.0316708 (PMC11805404; doi:10.1371/journal.pone.0316708)

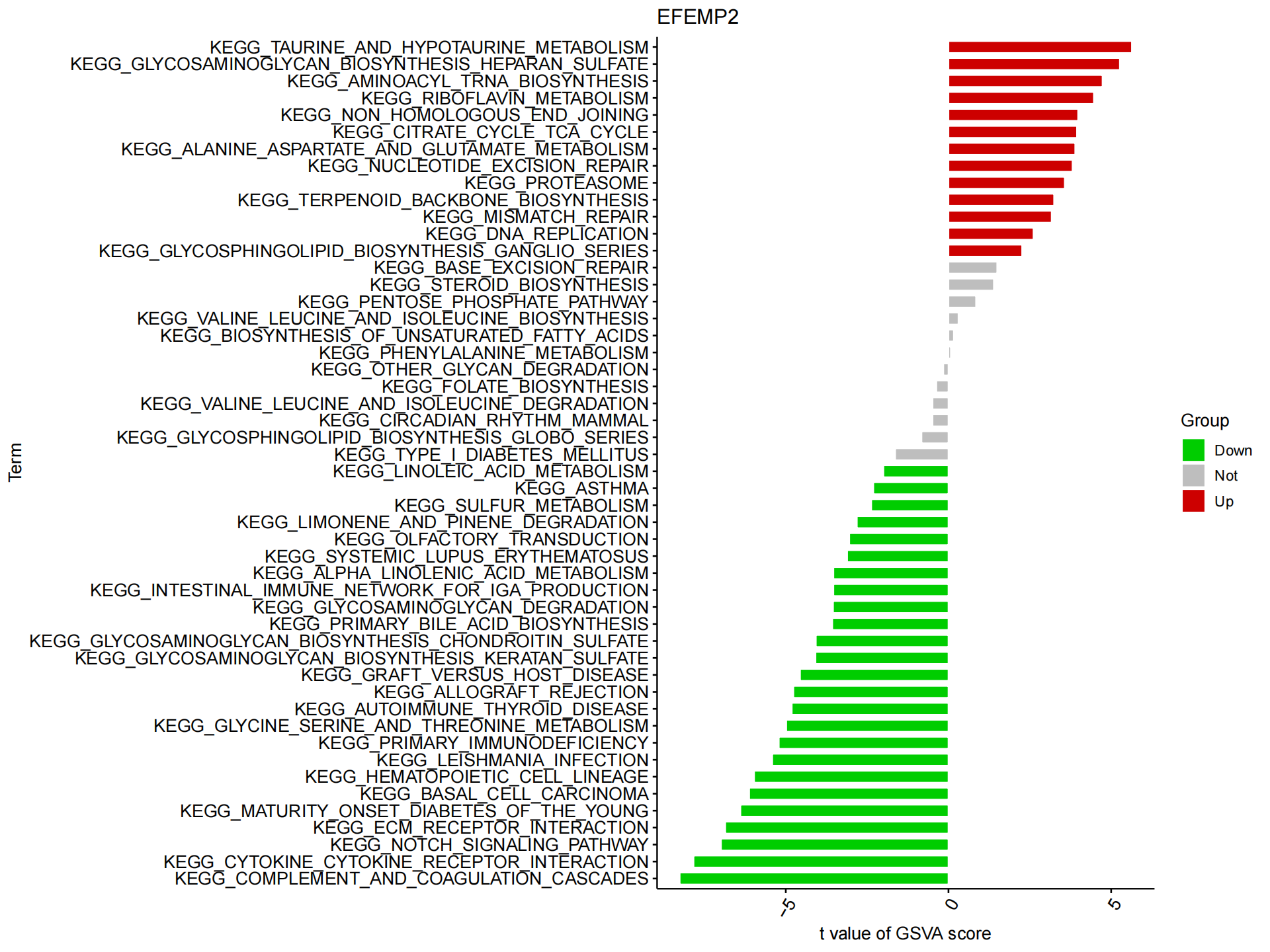

Supplement: S1 Fig — The x-axis represents the t-value of the GSVA score, which measures the degree of difference in expression levels of the core gene set between the disease group and the control group. A larger absolute value of the t-value indicates a more significant difference between the two groups. The y-axis lists different signaling pathways, which are collections of interconnected molecular events in biology. In the figure, red represents a positive t-value, indicating that the expression level of the gene set in the disease group is upregulated compared to the control group; whereas green represents a negative t-value of the GSVA score, indicating that the expression level of the gene set in the disease group is downregulated compared to the control group. (TIFF) [file pone.0316708.s012.tiff]

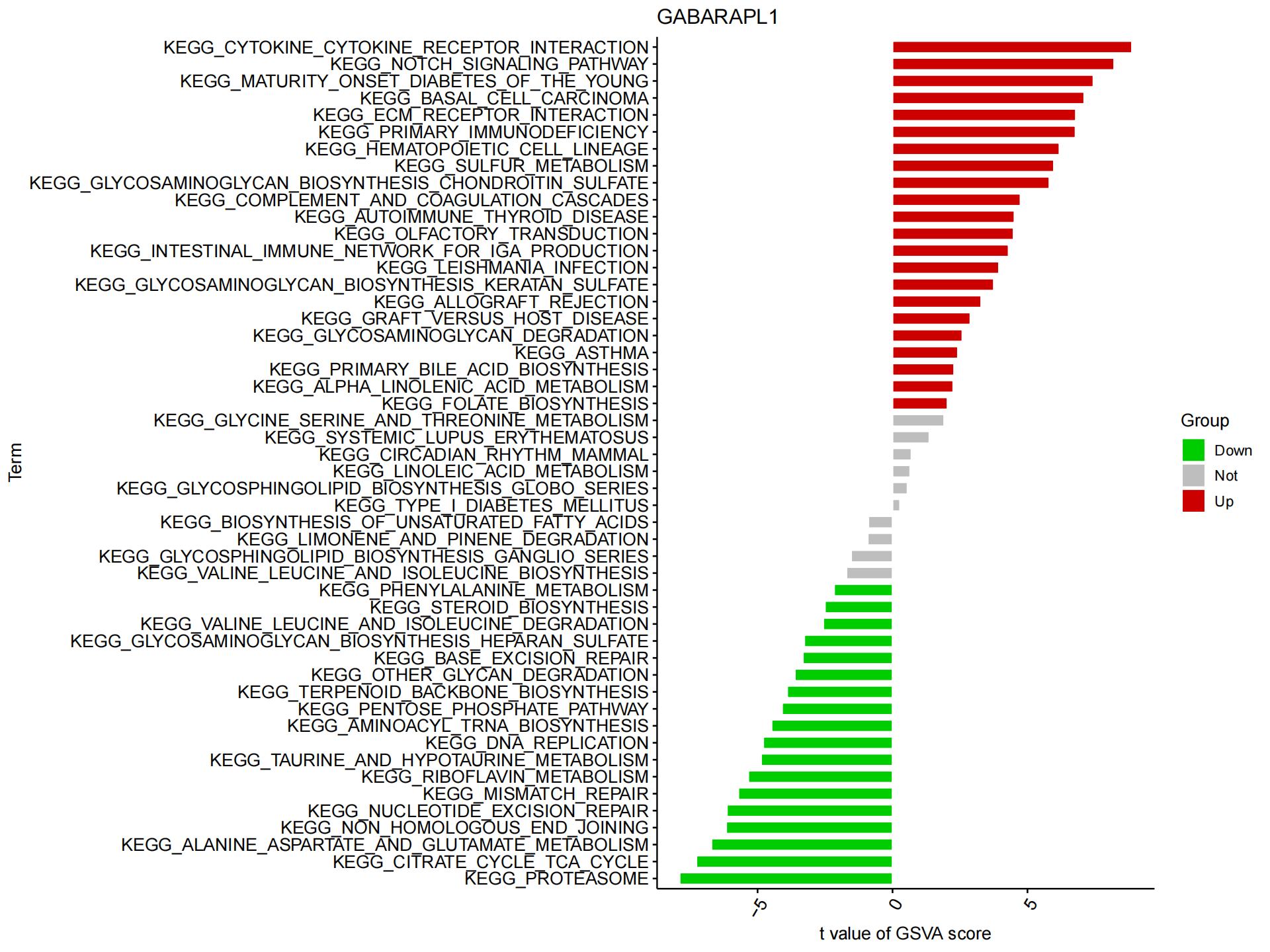

Supplement: S2 Fig — The x-axis represents the t-value of the GSVA score, which measures the degree of difference in expression levels of the core gene set between the disease group and the control group. A larger absolute value of the t-value indicates a more significant difference between the two groups. The y-axis lists different signaling pathways, which are collections of interconnected molecular events in biology. In the figure, red represents a positive t-value, indicating that the expression level of the gene set in the disease group is upregulated compared to the control group; whereas green represents a negative t-value of the GSVA score, indicating that the expression level of the gene set in the disease group is downregulated compared to the control group. (TIFF) [file pone.0316708.s013.tiff]

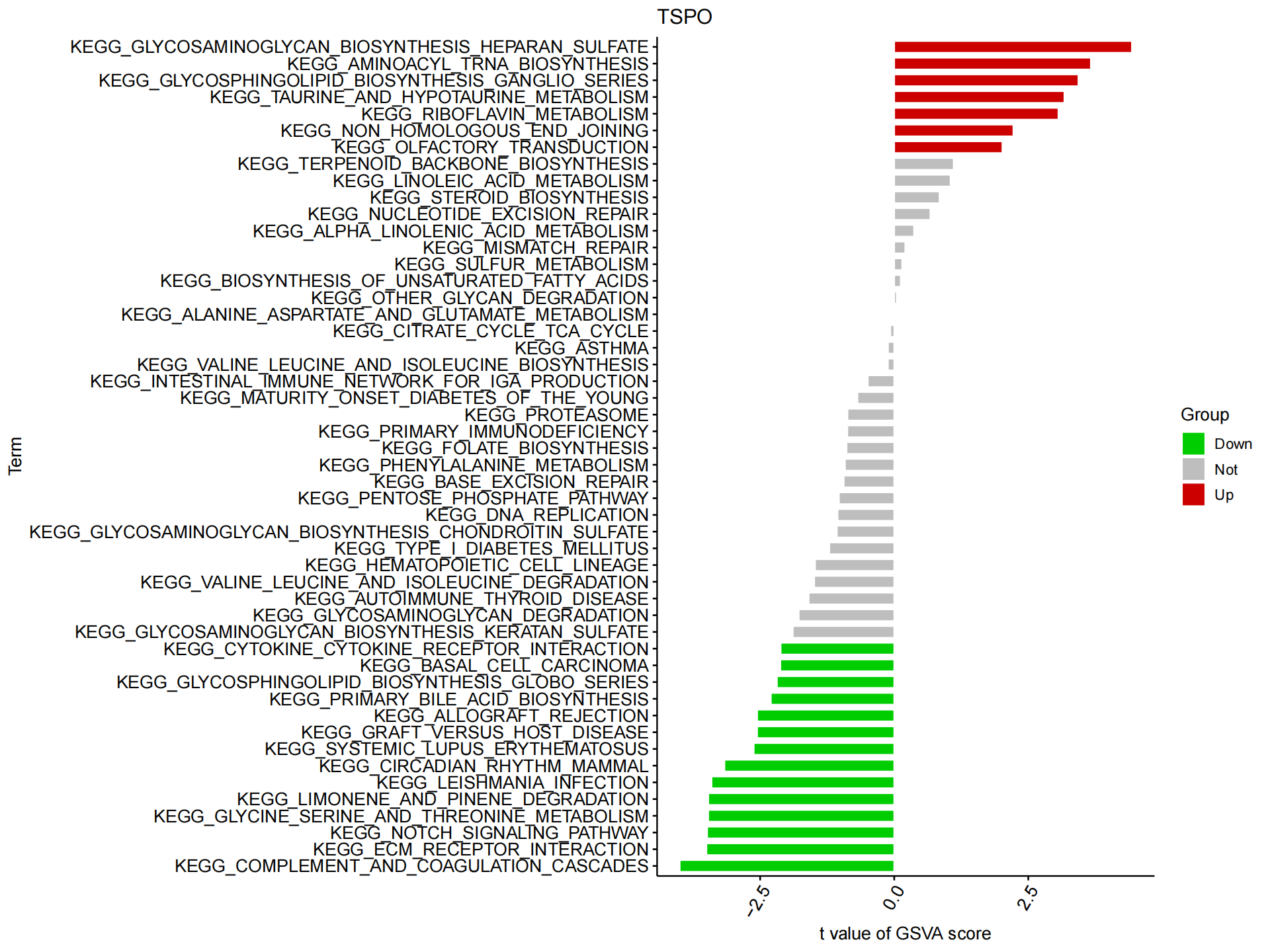

Supplement: S3 Fig — The x-axis represents the t-value of the GSVA score, which measures the degree of difference in expression levels of the core gene set between the disease group and the control group. A larger absolute value of the t-value indicates a more significant difference between the two groups. The y-axis lists different signaling pathways, which are collections of interconnected molecular events in biology. In the figure, red represents a positive t-value, indicating that the expression level of the gene set in the disease group is upregulated compared to the control group; whereas green represents a negative t-value of the GSVA score, indicating that the expression level of the gene set in the disease group is downregulated compared to the control group. (TIFF) [file pone.0316708.s014.tiff]

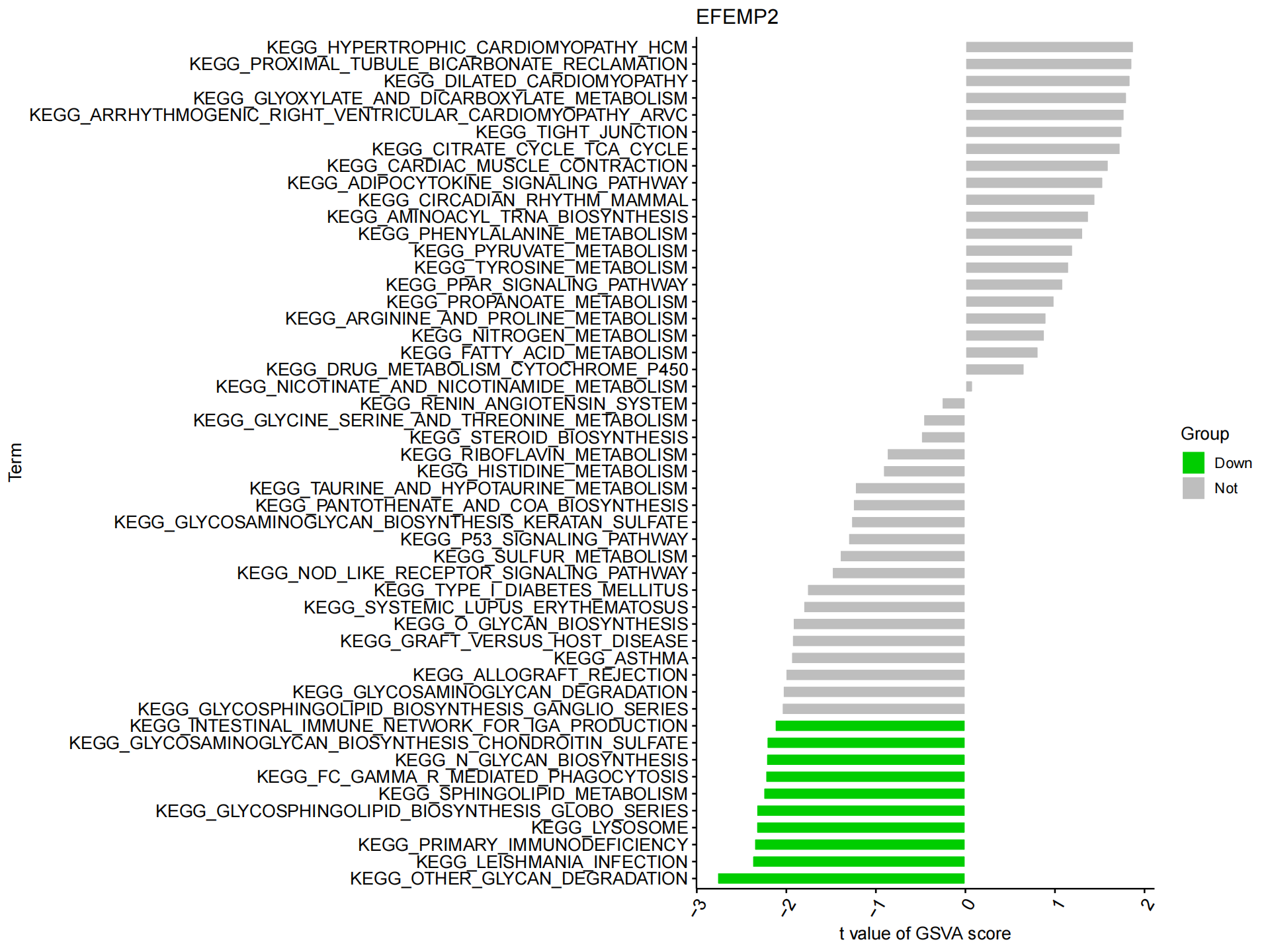

Supplement: S4 Fig — The x-axis represents the t-value of the GSVA score, which measures the degree of difference in expression levels of the core gene set between the disease group and the control group. A larger absolute value of the t-value indicates a more significant difference between the two groups. The y-axis lists different signaling pathways, which are collections of interconnected molecular events in biology. In the figure, red represents a positive t-value, indicating that the expression level of the gene set in the disease group is upregulated compared to the control group; whereas green represents a negative t-value of the GSVA score, indicating that the expression level of the gene set in the disease group is downregulated compared to the control group. (TIFF) [file pone.0316708.s015.tiff]

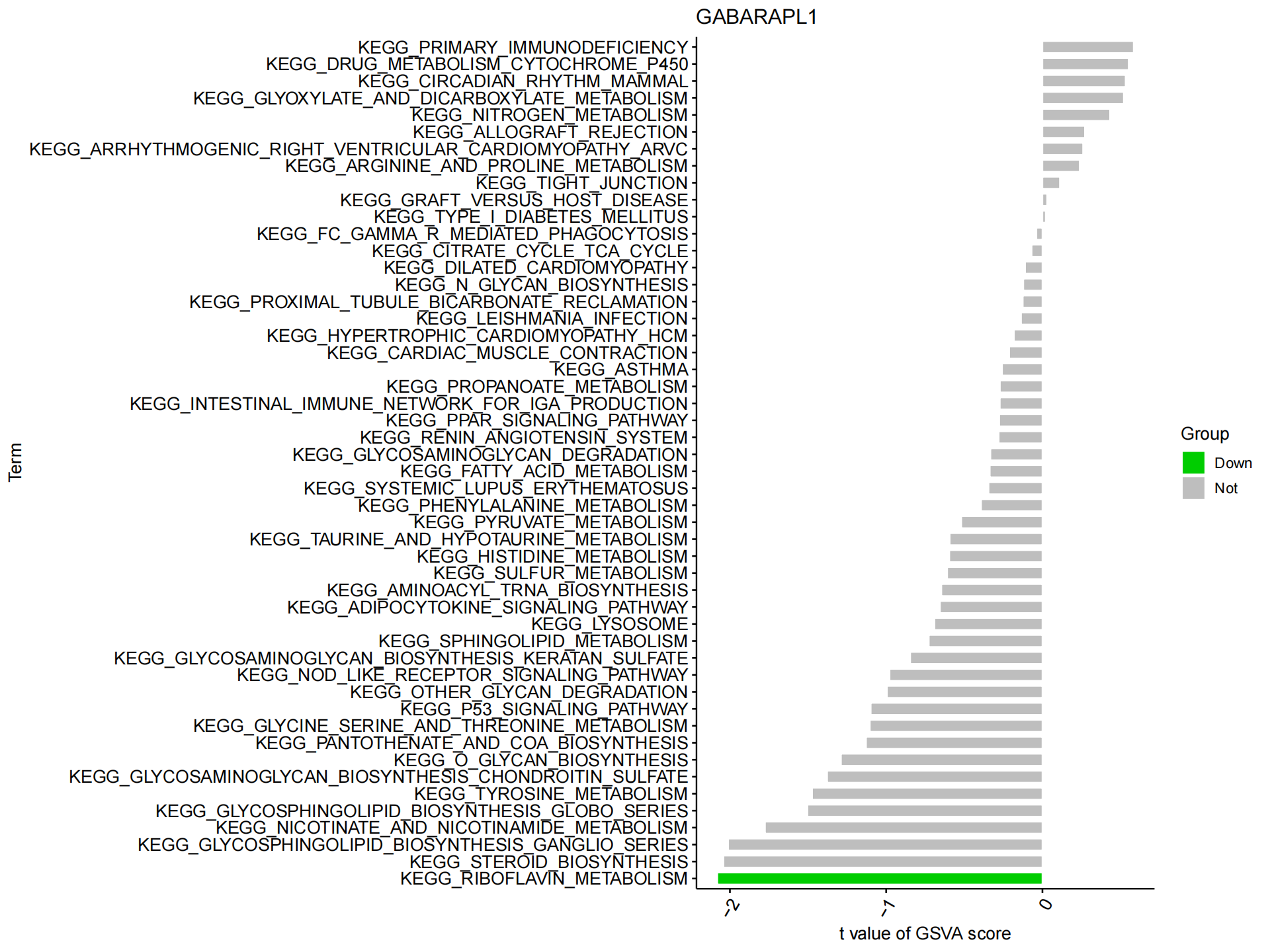

Supplement: S5 Fig — The x-axis represents the t-value of the GSVA score, which measures the degree of difference in expression levels of the core gene set between the disease group and the control group. A larger absolute value of the t-value indicates a more significant difference between the two groups. The y-axis lists different signaling pathways, which are collections of interconnected molecular events in biology. In the figure, red represents a positive t-value, indicating that the expression level of the gene set in the disease group is upregulated compared to the control group; whereas green represents a negative t-value of the GSVA score, indicating that the expression level of the gene set in the disease group is downregulated compared to the control group. (TIFF) [file pone.0316708.s016.tiff]

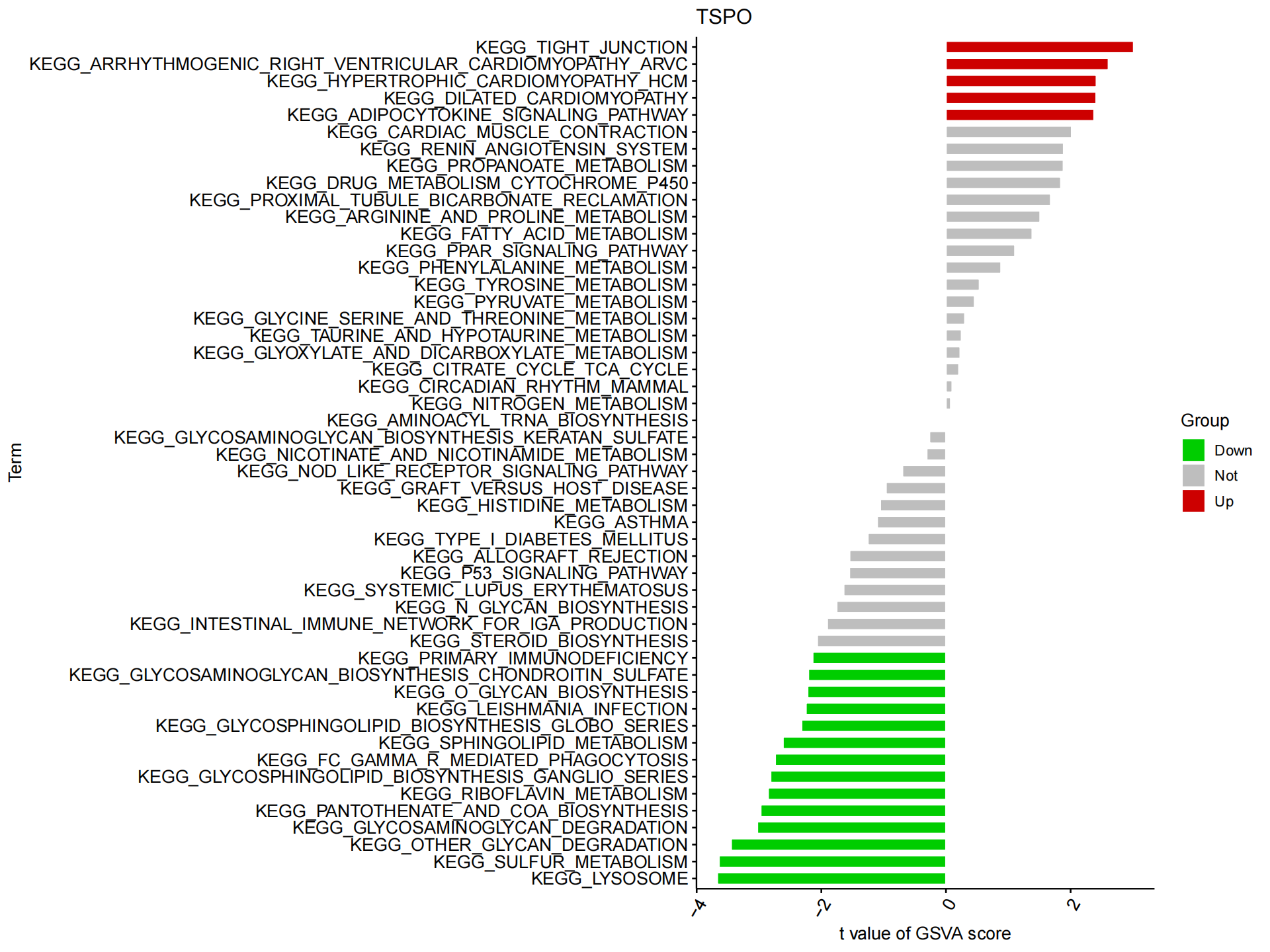

Supplement: S6 Fig — The x-axis represents the t-value of the GSVA score, which measures the degree of difference in expression levels of the core gene set between the disease group and the control group. A larger absolute value of the t-value indicates a more significant difference between the two groups. The y-axis lists different signaling pathways, which are collections of interconnected molecular events in biology. In the figure, red represents a positive t-value, indicating that the expression level of the gene set in the disease group is upregulated compared to the control group; whereas green represents a negative t-value of the GSVA score, indicating that the expression level of the gene set in the disease group is downregulated compared to the control group. (TIFF) [file pone.0316708.s017.tiff]
